# Transcriptional modulation of HIV-1C LTR promoter

**DOI:** 10.1186/1471-2334-12-S1-O6

**Published:** 2012-05-04

**Authors:** Anand Singh, Pradeep Ramalingam, Jayanth Kumar Palanichamy, Mohita Bhagat, Muzaffer Ahmad Kassab, Subrata Sinha, Parthaprasad Chattopadhyay

**Affiliations:** 1 All India Institute of Medical Sciences (AIIMS), New Delhi, India

## Background

All current anti-HIV1 therapies target the viral proteins or RNA; however targeting HIV1 at the transcriptional level of the integrated provirus has been less explored. In India, AIDS is commonly caused by HIV-1C compared to HIV-1B in developed countries. HIV1-5’LTR acts as a promoter and shows sequence variation among different clades. Transcriptional gene silencing (TGS) is a method wherein dsRNA targeting the promoter/enhancer of a gene are used to down regulate its expression.

## Methods

We used SiHa cell line stably expressing a bi-cistronic reporter system (5’LTR-SEAP-IRES-EGFP), in which secreted alkaline phosphatase (SEAP) and enhanced green fluorescent protein (EGFP) are expressed under 5’LTR of HIV-1B/C. The cell line was transfected with different dsRNAs (S1-S6) targeting the core promoter/enhancer of HIV-1C LTR to induce TGS. Screening for decreased transcription was done using real-time PCR (mRNA expression of SEAP and EGFP), fluorescence microscopy (EGFP) and flow cytometry (EGFP).

## Results

After single or multiple (thrice) transfection of dsRNAs, we identified one dsRNA (S4) which showed consistent and significant down regulation of both SEAP (44% & 68% respectively) and EGFP (40% & 65%) (p<0.001 in both cases) mRNA levels. This reporter down regulation was also confirmed by studying EGFP expression using fluorescence microscopy and flowcytometry which also showed a significant fall after S4 transfection.

## Conclusion

TGS usually involves epigenetic modifications like DNA methylation/histone methylation at the targeted region and induces long term suppression of gene expression. So targeting of the HIV-1C LTR by dsRNA can be used as a therapeutic modality in the future.

